# Assessment of Infestation of Selected Blackcurrant (*Ribes nigrum* L.) Genotypes by the Blackcurrant Leaf Midge (*Dasineura tetensi* Rübs.) in Poland

**DOI:** 10.3390/insects12060492

**Published:** 2021-05-25

**Authors:** Wojciech Piotrowski, Barbara H. Łabanowska, Marcin Kozak

**Affiliations:** 1 Laboratory of Entomology, Department of Plant Protection, National Institute of Horticultural Research, Konstytucji 3 Maja 1/3, 96-100 Skierniewice, Poland; basia.labanowska@gmail.com; 2 Department of Media, Journalism and Social Communication, University of Information Technology and Management, Sucharskiego 2, 35-225 Rzeszów, Poland; nyggus@gmail.com

**Keywords:** *Dasineura tetensi*, leaf curling midge, Cecidomyiidae, blackcurrant, genotype assessment

## Abstract

**Simple Summary:**

*Dasineura tetensi* is a widespread blackcurrant pest. The susceptibility of blackcurrant genotypes to leaf midge colonization was assessed to select the genotypes least susceptible to be used as parents in the breeding. Experiments were conducted between 2012–2014 in Poland. Percentage shoot damage, and number of eggs and larvae were assessed for each genotype. Among tested blackcurrant genotypes, none were found to be completely resistant to this pest. However, some genotypes (Big Ben, Nr 8/72, Ben Connan, Ben Alder, Ben Hope, Foxendown, Ben Nevis, Fariegh, Ojebyn, and Ben Tirran) were colonized by the pest below the threshold level (10%); proving tolerance to the pest. The fewest pest eggs were recorded on the genotypes Big Ben, Ben Connan, Ben Alder, and Ben Nevis, and out of these Big Ben, Nr 8/72, Ben Connan, and Foxendown had the least larvae recorded.

**Abstract:**

Blackcurrant leaf midge (*Dasineura tetensi*) is a widespread pest of blackcurrant. Attacks by this pest can cause up to 60% reduction in the growth of shoots resulting in yield decreases. Our study, conducted (2012–2014) in Poland, aimed to assess the susceptibility of blackcurrant genotypes to *D. tetensi*, in order to select genotypes as parental lines for breeding new blackcurrant genotypes. Among tested blackcurrant genotypes, none were found to be completely resistant to this pest. The pest colonized genotypes Big Ben, Nr 8/72, Ben Connan, Ben Alder, Ben Hope, Foxendown, Ben Nevis, Fariegh, Ojebyn, and Ben Tirran below the threshold level (10%). In contrast, genotypes Nr 7/15, Ben Lomond, Ben Finlay, Tisel, Polares, Polonus, Tiben, PC-110, Polben, Gofert, Ruben, and Ores suffered pest levels above the threshold. With regard to egg numbers, the fewest were recorded on genotypes Big Ben, Ben Connan, Ben Alder, and Ben Nevis, and the most on Gofert and Ores. Fewer larvae were recorded on genotypes Big Ben, Nr 8/72, Ben Connan, and Foxendown compared to Ben Lomond, PC-110, Gofert, Tiben, Polben, and Ores. Developing blackcurrant genotype resistance to leaf midge strongly supports the IPM strategy.

## 1. Introduction

Blackcurrant (*Ribes nigrum* L.) is an important fruit-growing crop [1,2,3]. It is grown on a large scale mainly in northern Europe and Russia [4], where it was domesticated at least 400 years ago [2]. Poland has been the largest producer of blackcurrant fruit in Europe for many years [5]. Berries are used in large-scale production of fruit juices and other processes where its high level of vitamin C and anthocyanins are highly valued [2,6]. Commercial blackcurrant fruit production in Poland, as in all other countries, is now completely mechanized [7]. 

Blackcurrant leaf midge, *Dasineura tetensi* Rübs. (Diptera: Cecidomyiidae), is a common blackcurrant pest in Poland, the United Kingdom (UK), Europe and New Zealand [4,8,9]. Its larvae constitute the most noticeable stage of this pest, which feed gregariously within the shelter of young, furled leaf galls at the shoot tips [10]. Blackcurrant is the only host of *D. tetensi* [11]. However, if midge larvae are transferred at the first instar stage they may gall and develop through to maturity on red currant (*R. rubrum*) [12,13]. Goncharova [14] showed that the salivary glands of *D. tetensi* contain amylase and proteolytic enzymes and that the mesophyll cells of the plant tissue fed on by the larvae suffered from cell lysis along with a reduction in chlorophyll content. Depending on the number of infesting larvae, part or the whole of the leaf becomes folded and twisted into a distinctive leaf gall. Older galls become necrotic. Several adjacent leaves on a shoot may be galled, with strong, vigorous shoots being most likely to be attacked [8,15,16]. In damaged blackcurrant leaves, the accumulation of flavonoids and hydroxycinnamic acids can be significantly lower than in undamaged leaves [9]. Galling damage also has a significant effect on the photosynthesis of blackcurrant bushes due to the assimilation area of leaves being reduced [10]. In addition, distorted young leaves can be contaminated by secondary fungal infestation [17], and leaf curling and twisting in a severe attack masks blackcurrant reversion virus (BRV) disease symptom (abnormal forms and patterns of leaves) which may prevent the recognition of rogue cultivars when inspecting nursery stocks [12]. 

The pest has two to four generations a year, with first generation oviposition occurring around the time of blackcurrant flowering, depending on the season and cultivar [13,15,18,19]. The first generation and start of emergence of the second generation are distinct, but later generations may overlap [15,20,21]. The midge overwinters as larvae within cocoons in the soil, which then pupate in early spring [8,15]. Shortly after emergence, mating occurs, and eggs are laid in the folds of very young leaves within the growing points of blackcurrant shoots [10]. Greenslade [22] found up to 14 eggs, but Hellqvist [13] recorded between 5–20 larvae on a single infested leaf, demonstrating that numbers can vary considerably. On average, usually 4–5 eggs are laid per leaf.

The blackcurrant leaf midge is most damaging to young establishing plantations, where severe infestation can lead to reductions in shoot growth, sometimes even leading to the complete destruction of the crop [4,23]; up to 90% of shoots can be damaged by heavy infestations of *D. tetensi* [24]. Within established crops, plants can counteract the damage by growing extra shoots and therefore control is less important than in the case of seedlings or in newly established plantations [8]. Greenslade [22], Barnes [12], and Łabanowska et al. [23] considered the midge as a serious pest of nursery stocks. Łabanowska et al. [23] reported that in mother plantations in Poland, *D. tetensi* attacks may cause up to 60% reduction in shoots growth, and that in nurseries, infested plants are weakened and do not grow to a suitable size because of the killing of terminals, causing a proliferation of side shoots from lateral buds. 

Stenseth [25] found in an experiment conducted in Norway investigating chemical control of *D. tetensi* that achieving control of the first and second generations resulted in a yield increase of approximately 50%. Reduction in the number of flowers per raceme and reduced fruit weight were considered to be responsible for most of the yield reduction due to midge attack. As broad-spectrum insecticides were used in their experiments, it is possible that other pests may also have been controlled so reducing overall impacts on the blackcurrant bushes. According to Goncharova [14], attack by *D. tetensi* in Russia frequently caused a yield reduction of nearly 25%. In comparison, Cross et al. [8] reported that in the UK, this pest had no effect on the quality and quantity of blackcurrant yield in established commercial plantations, but the average age of plantations in their work was more than eight years.

In Poland, *D. tetensi* was a serious pest in the seventies of the 20th century. After that, this pest was not considered an important pest until 2007. Until then it was possible to control it with broad spectrum insecticides. After the withdrawal of the last organophosphate agents from the blackcurrant plant protection program in 2007, populations of blackcurrant leaf midge increased. Currently Polish blackcurrant growers can use neonicotinoid insecticides containing acetamiprid and since 2020 spirotetramat for controlling the pest. Unlike growers in the UK [26], Polish blackcurrant growers do not have access to sex pheromone traps, and so they control the pest by two insecticide sprays (one for each generation), based on the appearance of first leaf damage in shoot tips and observation of larvae in curled (youngest) leaves. The threshold level is 10% of shoot tips damaged with larvae present in galls. However, it has been shown that insecticides containing acetamiprid give only partial control of *D. tetensi* [8]. Spirotetramat is very effective in controlling midge larvae, but this active ingredient and its metabolites, although below the EU maximum residue level (MRL), can be detected in blackcurrant fruit [7]. Therefore, this substance cannot be used on blackcurrant plantations with a ‘zero residues’ level requirement. 

Increasing consumer pressure to reduce residues on fruit and concerns about midge pesticide resistance calls for further work into the control of midges with a reduction of pesticides. One of the methods to achieve this goal in an IPM system is developing cultivars resistant to the pest [4]. Variation among blackcurrant genotypes in susceptibility to gall midge attack was recognised many years ago by Greenslade [22], Stenseth [25], North [27], Łabanowska [11], Keep [20], Hellqvist and Larsson [21], and Crook [28]. Some genotypes show antibiotic resistance to the midge, which is controlled by a dominant gene, Dt, identified in *Ribes dikuscha* [20], although its mode of action remains unclear. The resistance ranges from no gall formation and high larval mortality, to complete gall formation with larvae that survive but develop more slowly [13,21]. However, females do not discriminate resistant genotypes during the ovipositing period [21,28]. Hellqvist and Larsson [21] speculated that shoot vigor or abiotic factors influenced the expression of resistance. 

Hellqvist [13] showed that the observed variation in resistance can be due to variation in virulence among midges through two strains of the midge that had been recorded in Sweden: a virulent midge adapted to the resistant host and a non-adapted avirulent midge. The virulent midge was also found on the susceptible host and appeared to perform equally well as the avirulent midge. The virulent strain was dominant despite most of the cultivars grown having a susceptible genotype. In Poland, there is no information concerning which strains of the midge are dominant, but the colonization assessment of the blackcurrant cultivars and genotypes by this pest was undertaken many years ago by Łabanowska [11]. Blackcurrant cultivars cultivated in the past differed in their susceptibility to colonization by the midge. Some cultivars, e.g., Baldwin, showed partial resistance to midge attack, while others, e.g., Daniels September, were susceptible. Łabanowska [29] suggested that blackcurrant cultivars in which the top leaves remain closed for long periods (slow development), e.g., cultivars belonging to Ben group, are more susceptible to colonization by the leaf midge. In contrast, the cultivars whose top leaves grow rapidly, like Ojebyn, are less susceptible to the colonization by this pest. However, the assortment of cultivated cultivars of blackcurrant has changed; recently, many new cultivars have been bred which are characterized by higher yields and longer shelf life of the fruits. However, their host plant resistance (HPR) level to the midge is unknown. 

This study aims to assess the susceptibility of blackcurrant genotypes to blackcurrant leaf midge colonization in Poland. Based on the obtained results, it will be possible to select genotypes that are least colonized by the pest and use them as parental lines when breeding new blackcurrant genotypes. Developing a blackcurrant leaf midge resistant genotype is by far much better than relying upon chemical sprays in terms of environmental protection, fruit safety as well as the cost of repeated chemical spraying. Furthermore, such an advancement strongly supports the IPM strategy.

## 2. Materials and Methods

### 2.1. Experimental Plots

The experiments were carried out between 2012–2014 in a blackcurrant collection trial plantation located at the National Institute of Horticultural Research (NIHR), Experimental Orchard (51°54′55.6″ N, 20°06′58.8″ E) in Dąbrowice near Skierniewice, central Poland. Around this plantation were gooseberry (*Ribes grossularia* L.), blueberry (*Vaccinium corymbosum* L.) plantations and small woodland. In the autumn of 2007, the plantation was established in a random block, with four replications of five plants per plot. The row spacing was 3.5 m and the plants were spaced 0.6 m apart in the row (12.6 m^2^/plot). In addition, the distance between plots was 1.2 m. The bushes had a height of about 1.0–1.5 m (depending on genotype), but in the autumn 2011, shoots were cut back by hand pruner to 15 cm in height from the ground to encourage new shoot growth the following spring for oviposition by female of *D. tetensi*. Re-growing cut-down plantations is common practice in Poland especially on these where the blackcurrant gall mite (*Cecidophyopsis ribis* Westw.) and blackcurrant reversion virus are not a problem. On plantations larger than 2.0 ha, this work is mechanized. Twenty-two blackcurrant genotypes were used in the experiment. They came from England, Poland, Scotland, and Sweden (Table 1). 

The mean annual rainfall during the experimental period was 600 mm, mainly concentrated during the spring and summer months, over a vegetation season between March and October. The experiment was established on a mineral soil, fawn floor of light clay with a pH of 6.0–6.5. The plantation had no irrigation or fertigation systems.

Blackcurrant plants received no chemical protection against pests and diseases. The plantation was fertilized and weeds were controlled with contact-acting herbicides according to recommendations for commercial IPM blackcurrant plantations.

### 2.2. Measurements and Observations

The genotypes were evaluated twice per year, one week after the first and second generations of *D. tetensi*. In these periods of time damage leaves on shoots were easy to found. In Poland, the first generation of the blackcurrant leaf midge usually occurs shortly after flowering (mid-May), and the second towards the end of June or early July. During each assessment, firstly, the percentage shoot damage was assessed by counting the total number of damaged and undamaged shoots on each bush in situ. Shoots with no leaf midge symptoms were classified as undamaged shoots, and shoots with leaf midge symptoms were classified as damaged shoots, regardless of whether it had slight, moderately or severe damaged. Afterwards, on each sampling occasion twenty vigorous shoots were taken per genotype (one from each bush). Samples were then placed in plastic bags and labeled, before being taken to the NIHR’s laboratory, where the shoots were dissected and examined for eggs and larvae under a stereo binocular microscope. The upper and lower surface of each young leaf (regardless of their location on shoot) was examined.

In 2012 shoots had a length of about 0.15–0.20 m (first evaluation), and about 0.40–0.50 m during the second evaluation, at the time of damage assessment and sampling for eggs and larvae. In 2013 shoots lengths were from 0.20 to 1.20 m, and in 2014 shoots had a length of between 0.20 and 1.5 m at the time of damage assessment. The length of shoots at the time of sampling for eggs and larvae was about 0.50–0.60 m in 2013 and 2014. 

### 2.3. Statistical Analysis

Keep [20] used the 1–5 scale to grade blackcurrant genotypes (younger than 10 years) in terms of *D. tetensi* infestation. Grade 1 indicated no or very slight leaf symptoms of attack; Grade 3 indicated that leaves on a few shoots only were moderately distorted; and Grade 5 represented very severe infestation, with all shoots carrying many severely distorted leaves. Hellqvist and Larsson [21] used the 0–6 scale to assess the degree colonization of blackcurrant genotypes by *D. tetensi*. Grade 0 indicated no leaf symptoms, while Grade 6 indicated very severe infestation, with all shoots having several completely distorted leaves. However, in modern times such scales are now not considered a precise tool for assessing sensitivity of blackcurrant genotypes to midge infestation, since they flatten infestation data. Several previous studies have screened blackcurrant genotypes by assessing damage (galls) and/or egg and larval numbers, but they concentrated only on one year [19] or included only one pest generation in a year, or compared well known susceptible and resistant genotypes [21]. However, there was a need to find a model which covered together three years study and both pest generations. To analyze the probabilities of shoot damage for the genotypes, we initially wanted to use generalized linear mixed effect modeling. The reasoning behind using generalized linear models was that the dependent variable was binary (a shoot was either damaged or not), and for using mixed effect models was the nesting of bushes within replications as well as within the following years. Since it makes little sense to compare the two generations, because the second generation is always more damaging than the first one, we decided to build separate models for them. Estimation in generalized linear mixed effects models is complex, so we additionally decided to simplify the models by the following approach. We aimed to compare probabilities of shoot damage. In one replication, we had five bushes, and of course we could not treat them independently. However, we summed all the shoots from these bushes (including damaged ones), and instead of calculating the probability per bush and then estimating the per-replication effect (which would require us to include random effects in the model), we did so for such overall data per replication (so, together for all the five bushes per replication). In other words, we worked with the number of damaged shoots per replication, not per bush. Such a procedure did not affect the per-replicate treatment and provided the very same interpretation, but at the same time, it aided us in simplifying the model, since we did not have to include the corresponding random effect. We could do so thanks to the complete balance of the data, with no bush having been removed during the course of the experiment; thus, the data were balanced at a replication level, with five bushes per replication. This led us to generalized linear mixed effects models, for each generation, with the only random effect being that which corresponded to the repeated measurements in the subsequent years. The resulting models (for both generations), however, were singular, making it impossible to fit them. Therefore, instead we built separate generalized linear models for each generation in each year, using the binomial distribution; thus, they did not require any random effect.

All the models showed significant effects of the genotype, an unsurprising and rather uninteresting result given the number of genotypes tested. Therefore, we based our interpretation on estimates of shoot damage probabilities for each genotype, in the three years and the two generations; we estimated them, along with the 95% confidence intervals, using the generalized linear models built. We checked the assumptions using graphical methods [30]. The final analysis used the standard glm function from the stats package R [31], while our attempts to fit generalized linear mixed effects models used the glmer function from the lme4 package R [32]. 

The number of larvae and eggs data were analyzed using general linear models. This time, there was no need for using random effects, since there was no nesting in the data.

## 3. Results

### 3.1. Response of Blackcurrant Genotypes to Leaf Midge (Dasineura tetensi) in the Field

The level of shoots damaged by *D. tetensi* was different on various genotypes. Among tested genotypes, none were found to be completely resistant to the pest. Probability of shoots damaged on blackcurrant genotypes by the larvae of *D. tetensi* (both pest generations) was lower than the threat threshold (10%) on the first 10 genotypes: Big Ben, Nr 8/72, Ben Connan, Ben Alder, Ben Hope, Foxendown, Ben Nevis, Fariegh, Ojebyn, and Ben Tirran. They can therefore be considered as tolerant genotypes to this pest. They grew normally although some leaves were damaged on them. However, the remaining 12 genotypes: Nr 7/15, Ben Lomond, Ben Finlay, Tisel, Polares, Polonus, Tiben, PC-110, Polben, Gofert, Ruben, and Ores, were more strongly damaged by the pest, all above the 10% threshold. This result suggests that it is not possible to rely on the use of genotypes from that group to manage the midge and therefore other pest control methods are required (Table 2).

### 3.2. Eggs Number 

The mean numbers of *D. tetensi* eggs found on different blackcurrant genotypes varied, ranging from 0.25 to 9.00. The fewest eggs were laid on genotype shoots belonging to Big Ben, Ben Connan, Ben Alder, and Ben Nevis. The most eggs were recorded on the shoots of genotypes Gofert and Ores (Table 3).

### 3.3. Larvae Number

Mean leaf midge larvae numbers varied between the genotypes, ranging from 0.25 to 10.50. Fewer *D. tetensi* larvae were found on the shoots of the genotypes Big Ben, Nr 8/72, Ben Connan, and Foxendown suggesting that likely occurrence of antibiosis mechanisms in them; more were recorded on the shoots of genotypes Ben Lomond, PC-110, Gofert, Tiben, Polben, and Ores. All of the larvae found were alive and varied in color and size, due to the fact that eggs had been laid at different times in the field (Table 4). 

## 4. Discussion

During 2012–2014, blackcurrant leaf midge infestations at the Experimental Orchard were so severe and widespread that it left no doubt that none of the blackcurrant genotypes grown there were fully resistant to this pest. In the past, some genotypes from northern Scandinavia (Sunderbyn II and Kangosfors) and Russia (e.g., Dikovinka) showed genetic resistance with no symptoms of attack [20], but our collection does not include them. The blackcurrant genotype Storklas which is resistant to midge attack was listed by Hellqvist and Larsson [21]. In greenhouse studies, Storklas displayed similar resistance to that of Sunderbyn II, that is, no ovipositional discrimination and high first larval instar mortality. However, resistance against *D. tetensi* in Storklas seems to be unstable; in Hellqvist and Larsson’s [21] study larval performance ranged from 100% mortality to successful though slow development. Similarly, in field experiments, Storklas showed almost no symptoms of galling in one field but a rather high degree in the other, despite similar infestation levels on susceptible genotypes in neighboring plots in both fields. According to Hellqvist [13], variation in larval performance on Storklas was at least in part, due to genetic variation in virulence among midges. That study concluded that at least two different biotypes (A and B) of *D. tetensi* existed in northern Sweden, the two being characterized by the ability, or its lack of, to develop on the resistant cultivar Storklas. Larvae of biotype A are avirulent on Storklas; they do not cause galling symptoms on the leaves and they do not develop into mature larvae. Those of biotype B are virulent on Storklas; larvae cause gall formation on the leaves and they develop to maturity. 

In Poland, *D. tetensi* was first detected near Skierniewice (Sochaczew) in 1958 by Stępniewska [33]. There is no information on which strains of the midge were dominant, but probably both biotypes were present, because none of the genotypes assessed were completely resistant. In our study, the genotypes Big Ben, Nr 8/72, Ben Connan, Ben Alder, Ben Hope, Foxendown, Ben Nevis, Fariegh, Ojebyn, and Ben Tirran were less colonized/tolerant (<10%) by *D. tetensi* than Nr 7/15, Ben Lomond, Ben Finlay, Tisel, Polares, Polonus, Tiben, PC-110, Polben, Gofert, Ruben, and Ores. Three of the 22 genotypes assessed in the field were also included in the study by Keep [20] in Scotland: Ben Nevis, Ben Lomond, and Ojebyn. Here, they were found to be highly susceptible to *D. tetensi*. Ojebyn was also susceptible to the leaf midge in trials led by Hellqvist and Larsson [21]. In our trials, however, only Ben Lomond was susceptible and Ben Nevis and Ojebyn were less infested. According to Brennan [2], Ben Connan is resistant while Ben Alder and Ben Tirran are susceptible to leaf midge. Our study did not confirm this, with all three of these genotypes falling into the group of being less colonized by the midge. Further along, Buczacki and Harris [34] reported that Ben Connan was partially resistant to leaf midge, an observation confirmed in our experiment. Crook et al. [19] also investigated susceptibility of blackcurrant genotypes to leaf midge, and in their field experiments, Ben Connan showed significantly smaller leaf curl than did Ben Alder. As was the case for Ben Connan, our work confirms their results, because for Ben Alder we received the opposite result. Griffiths et al. [1] analyzed leaves of blackcurrant genotypes susceptible and resistant to the midge using gas-chromatography linked to mass spectrometry. They found that the chemical make-up of odor plumes produced by two susceptible genotypes (Ben Alder and Ben Tirran) and one resistant genotype (Ben Connan) were identical, with only small quantitative differences between the three genotypes. There were no clear trends, with one volatile present in higher concentration in resistant versus susceptible plants or vice versa. These results are in line with ours, because in the current study all the three blackcurrant genotypes showed similar resistance (and were only a little susceptible to the pest). In addition, a search of the available literature did not expose any other research on blackcurrant leaf midge utilizing these genotypes as in our study.

Crook and Mordue [17] reported that adult mated females of *D. tetensi* responded to blackcurrant odor emitted from leaves and buds, suggesting that olfaction plays a crucial role in finding a suitable oviposition site. Unmated females were not attracted to host-plant odor stimulus, suggesting that mating is a trigger for a switch in behavior and is a pre-requisite to searching for an oviposition site. Crook et al. [19] investigated midge oviposition on blackcurrant plants of both resistant and susceptible cultivars. Single mated females, which were less than twenty-four hours old, were introduced to shoots of either Ben Alder (susceptible) or Ben Connan (midge resistant). Twenty-four hours later, the shoots were examined under a microscope for eggs. The females showed no preference towards any of the two genotypes. Genotypes Ben Alder and Ben Connan were also investigated within our study, and we achieved similar results, because only a few midge eggs were found on these genotypes. According to Hellqvist and Larsson [21], host acceptance by *D. tetensi* females may be influenced by egg load; that is, ovipositing females may be less discriminative when having many eggs in their abdomen than those that carry fewer eggs. If this was the case, females should have been choosier during the second ovipositional period, by which time they had already deposited some of their eggs. However, they found no indication of such a change in motivation to oviposit between periods, further supporting the hypothesis that *D. tetensi* females do not discriminate against resistant plants. The differences in the number of eggs laid by *D. tetensi* on susceptible (Ojebyn) and resistant (Storklas) genotypes were not significant. In our study, we also did not see differences in the number of eggs laid on Ojebyn and Big Ben, Ben Connan, Ben Adler, and Ben Nevis. Female antixenosis is not the main mechanism for resistance to *D. tetensi* on resistant genotypes. In our study, besides Ben Connan and Ben Alder, the fewest number of eggs were also found on genotypes Big Ben, Ben Alder, Ben Nevis, and the most eggs were recorded on Gofert and Ores genotypes. However, we are not aware of any obvious or consistent association between plant and insect between tolerant and susceptible blackcurrant genotypes that females could use as ovipositional cues.

Crook et al. [19] observed in the field study that larvae reared on Ben Connan (midge-resistant) plants were smaller in size than those reared on Ben Alder (midge-susceptible), but no statistical analysis was performed, due to the fact that larvae were hatched at different times in the field. However, in a controlled conditions study, they archived that, larvae reared on the susceptible cv. Ben Alder were significantly longer than on Ben Connan. We did not observe such differences, because we assessed the genotypes only twice each year, but parameters such as larval size or weight would be a valuable metric to measure in future investigations; alternatively, fewer larvae were found on Ben Connan than on Ben Alder. There were large differences in larval growth between the two blackcurrant genotypes Ojebyn (susceptible) and Storklas (resistant). In some replicates, larvae did not increase in size and remained in their first instar until they died on resistant genotype. Larvae did grow on other replicates, although much more slowly than on susceptible genotypes [21]. This result is similar to ours, because on genotype Ojebyn more larvae were found than on Big Ben, Nr 8/72, Ben Connan, and Foxendown. The fact that fewer larvae were found on shoots of some genotypes, suggests that larval antibiosis during the larva–hostplant interactions at gall formation may be an important factor in plant resistance in *D. tetensi*. 

Hellqvist and Larsson [21] claimed that for larval performance, the manipulation of the host leaf appears crucial. Slow gall initiation translates into greater leaf expansion before the larvae affect it, so in this way the leaf suffers less damage. However, larval development may be influenced by plant vigour [8] and direct abiotic factors such as temperature or humidity [35]. In the field, where the pest needs to struggle with various stressful conditions, it may be important for the larvae to quickly transform the leaf into a gall (independently of blackcurrant genotypes), where they would be more sheltered [13].

## 5. Conclusions

Among the blackcurrant genotypes tested, none were fully resistant to *Dasineura tetensi*. However, our survey showed that there are some genotypes such as Big Ben, Nr 8/72, Ben Connan, Ben Alder, Ben Hope, Foxendown, Ben Nevis, Fariegh, Ojebyn, and Ben Tirran, which were colonized by the pest below the threshold level of 10%. They can therefore be considered as tolerant genotypes to this pest, and potentially be used as parental lines when breeding new blackcurrant genotypes. These genotypes are recommended to planting on plantations with an IPM system. The study also demonstrated that other genotypes Nr 7/15, Ben Lomond, Ben Finlay, Tisel, Polares, Polonus, Tiben, PC-110, Polben, Gofert, Ruben, and Ores were damaged above the threshold level. This suggests that it is not possible to rely only on them to manage midge populations and that other pest control methods—e.g., chemical spraying—are required. In addition, fewest eggs of the pest were laid on genotypes Big Ben, Ben Connan, Ben Alder, and Ben Nevis, and most eggs were found on genotypes Gofert and Ores. Furthermore, fewer *D. tetensi* larvae were found on the shoots of genotypes Big Ben, Nr 8/72, Ben Connan, and Foxendown, and more larvae were recorded on the shoots of genotypes Ben Lomond, PC-110, Gofert, Tiben, Polben, and Ores. This research will be continued in the new blackcurrant collection trial by the National Institute of Horticultural Research.

## Figures and Tables

**Table 1:** List and origin of blackcurrant genotypes evaluated in the trials.

No.	Genotype	Country of Origin	Parental Lines
1	Farliegh	England	BC_2_ *Ribes bracteosum* × BC_3_ from gooseberry
2	Foxendown	England	Ben Lomond × (BC_3_ from gooseberry × BC_2_ *R. glutinosum*)
3	Tisel	Poland	Titania × self-pollinated
4	Tiben	Poland	Titania × Ben Nevis
5	Ores	Poland	(Ojebyn × S_24_) × Ceres
6	Ruben	Poland	Biełoruskaja Słodkaja × Ben Lomond
7	Polonus	Poland	(C2/1/62 × Ben Alder) × EM B1834/145
8	Polares	Poland	S12/3/83 × EM B1834/113
9	Nr 7/15	Poland	Unknown
10	Nr 8/72	Poland	Unknown
11	PC-110	Poland	Ojebyn × Bieloruskaja Slodkaja
12	Polben	Poland	Ben Lomond × C2/1/62
13	Gofert	Poland	Gołubka × Fertodi-1
14	Ben Lomond	Scotland	(Consort × Magnus) × (Brodtorp × Janslunda)
15	Ben Nevis	Scotland	(Consort × Magnus) × (Brodtorp × Janslunda)
16	Ben Alder	Scotland	Ben More × Ben Lomond
17	Big Ben	Scotland	(Goliath × Ojebyn) op × (Ben Nevis × Vistavotchnaja)
18	Ben Connan	Scotland	Ben Sarek × Ben Lomond
19	Ben Tirran	Scotland	Ben Lomond × [(Seabrooks Black × Amos Black) × (Seabrooks Black × *Ribes* sp.)]
20	Ben Hope	Scotland	Westra × (238/36 × EM21/15)
21	Ben Finlay	Scotland	[(SCRI P10/9/13 × Ben Alder) × EM B1834-67]
22	Ojebyn	Sweden	Unknown

**Table 2:** Probability of shoot damage per genotype. The cells show estimates with 95% confidence intervals estimated using generalized linear models. The genotypes are sorted according to the increasing mean probability across the three years and both pest generations.

Genotype	Estimated Probability of Damage of a Shoot (Estimates with 95% Confidence Intervals)					
	First Generation			Second Generation		
	2012	2013	2014	2012	2013	2014
Big Ben	0.001 0.010 0.046	0.003 0.018 0.057	0.000 0.008 0.034	0.012 0.038 0.092	0.010 0.033 0.078	0.012 0.034 0.075
Nr 8/72	0.002 0.012 0.039	0.002 0.011 0.034	0.006 0.020 0.046	0.014 0.035 0.072	0.019 0.042 0.079	0.016 0.036 0.069
Ben Connan	0.001 0.009 0.041	0.000 0.008 0.034	0.005 0.019 0.051	0.019 0.048 0.100	0.020 0.047 0.093	0.024 0.051 0.094
Ben Alder	0.006 0.023 0.060	0.002 0.012 0.038	0.004 0.016 0.042	0.030 0.062 0.115	0.032 0.062 0.109	0.035 0.064 0.107
Ben Hope	0.008 0.026 0.061	0.010 0.029 0.064	0.004 0.016 0.042	0.014 0.037 0.076	0.040 0.073 0.123	0.032 0.060 0.103
Foxendown	0.007 0.028 0.073	0.006 0.024 0.064	0.005 0.022 0.058	0.011 0.035 0.083	0.038 0.078 0.141	0.025 0.054 0.104
Ben Nevis	0.006 0.025 0.066	0.005 0.020 0.052	0.004 0.016 0.042	0.022 0.052 0.103	0.043 0.079 0.134	0.030 0.057 0.098
Fariegh	0.012 0.038 0.090	0.010 0.034 0.080	0.013 0.038 0.083	0.026 0.063 0.124	0.024 0.056 0.112	0.021 0.050 0.100
Ojebyn	0.015 0.038 0.078	0.020 0.041 0.075	0.011 0.025 0.050	0.033 0.063 0.108	0.020 0.041 0.073	0.052 0.081 0.120
Ben Tirran	0.019 0.048 0.100	0.020 0.047 0.092	0.007 0.022 0.052	0.031 0.067 0.123	0.029 0.059 0.105	0.047 0.081 0.130
Nr 7/15	0.021 0.055 0.114	0.019 0.049 0.101	0.012 0.034 0.074	0.055 0.104 0.181	0.069 0.120 0.196	0.082 0.133 0.205
Ben Lomond	0.010 0.032 0.075	0.007 0.023 0.054	0.014 0.033 0.066	0.042 0.080 0.138	0.099 0.149 0.217	0.152 0.210 0.283
Ben Finlay	0.031 0.064 0.114	0.035 0.066 0.113	0.037 0.067 0.111	0.060 0.102 0.163	0.072 0.115 0.174	0.110 0.159 0.223
Tisel	0.002 0.009 0.029	0.010 0.025 0.052	0.019 0.037 0.066	0.067 0.105 0.156	0.124 0.173 0.236	0.187 0.243 0.313
Polares	0.016 0.046 0.102	0.024 0.056 0.112	0.013 0.035 0.078	0.124 0.198 0.304	0.086 0.144 0.227	0.150 0.223 0.321
Polonus	– 0.000 ^#^ –	0.001 0.008 0.035	0.002 0.014 0.043	0.189 0.282 0.410	0.198 0.287 0.405	0.217 0.303 0.417
Tiben	0.010 0.028 0.061	0.017 0.038 0.073	0.017 0.036 0.066	0.204 0.279 0.375	0.248 0.323 0.417	0.222 0.287 0.366
PC-110	0.082 0.128 0.192	0.103 0.151 0.214	0.119 0.167 0.228	0.132 0.189 0.264	0.238 0.312 0.403	0.299 0.376 0.468
Polben	0.016 0.041 0.084	0.023 0.049 0.091	0.029 0.055 0.094	0.348 0.460 0.602	0.403 0.512 0.648	0.431 0.536 0.665
Gofert	0.004 0.016 0.043	0.025 0.049 0.085	0.041 0.068 0.107	0.194 0.265 0.354	0.374 0.469 0.584	0.744 0.880 1.041
Ruben	0.002 0.015 0.046	0.002 0.013 0.041	0.002 0.011 0.036	0.545 0.703 0.905	0.546 0.695 0.882	0.587 0.734 0.915
Ores	0.012 0.032 0.065	0.016 0.036 0.068	0.017 0.036 0.065	0.553 0.686 0.848	0.579 0.705 0.858	0.683 0.814 0.969

^#^ For genotype Polonus in 2012 in the first generation, no shoots were damaged, so this scenario was removed from the statistical analysis.

**Table 3:** Estimated mean number of eggs from four five-shoot replications per genotype. The cells show estimates with 95% confidence intervals estimated using generalized linear models. The genotypes are sorted according to the increasing mean probability across the three years and both pest generations.

Genotype	Estimated Mean Number of Eggs per Five-Shoot Replication (Estimates with 95% Confidence Intervals)					
	First Generation			Second Generation		
	2012	2013	2014	2012	2013	2014
Big Ben	0.09 0.75 2.66	0.05 0.50 1.81	0.20 1.00 2.86	1.05 2.25 4.13	0.14 0.75 2.20	1.84 3.25 5.23
Ben Connan	0.00 0.25 1.98	0.00 ^#^	0.69 2.00 4.38	1.57 3.00 5.11	0.06 0.50 1.77	1.66 3.00 4.92
Ben Adler	1.00 ^#^	0.00 ^#^	0.56 1.75 4.01	1.39 2.75 4.79	0.65 1.75 3.70	1.12 2.25 3.95
Ben Nevis	0.03 0.50 2.20	0.05 0.50 1.81	1.30 3.00 5.78	0.89 2.00 3.79	0.65 1.75 3.70	2.03 3.50 5.55
Fariegh	1.00 ^#^	0.01 0.25 1.34	0.84 2.25 4.73	1.22 2.50 4.46	0.06 0.50 1.77	3.19 5.00 7.39
Foxendown	0.20 1.25 3.49	0.24 1.00 2.64	2.13 4.25 7.44	0.57 1.50 3.10	0.14 0.75 2.20	1.48 2.75 4.60
Ben Tirran	0.37 1.50 3.89	0.05 0.50 1.81	0.84 2.25 4.73	2.30 4.00 6.38	0.37 1.25 2.97	1.84 3.25 5.23
Nr 8/72	0.49 1.75 4.27	0.00 ^#^	3.96 6.75 10.62	0.73 1.75 3.45	1.11 2.50 4.74	0.02 0.25 1.04
Polares	0.49 1.75 4.27	0.14 0.75 2.24	0.99 2.50 5.09	1.75 3.25 5.43	0.14 0.75 2.20	2.41 4.00 6.17
Ojebyn	1.00 ^#^	0.49 1.50 3.39	1.79 3.75 6.78	1.39 2.75 4.79	0.65 1.75 3.70	1.66 3.00 4.92
Ben Finlay	0.37 1.50 3.89	0.36 1.25 3.02	1.30 3.00 5.78	1.39 2.75 4.79	0.37 1.25 2.97	2.60 4.25 6.47
Ruben	0.37 1.50 3.89	0.14 0.75 2.24	1.14 2.75 5.43	2.68 4.50 7.00	0.37 1.25 2.97	2.80 4.50 6.78
Polben	0.26 1.25 3.49	0.49 1.50 3.39	1.30 3.00 5.78	1.39 2.75 4.79	2.14 4.00 6.71	1.84 3.25 5.23
Ben Lomond	1.04 2.75 5.72	0.36 1.25 3.02	1.14 2.75 5.43	1.93 3.50 5.75	0.37 1.25 2.97	3.00 4.75 7.08
Ben Hope	0.76 2.25 5.01	0.36 1.25 3.02	1.14 2.75 5.43	2.30 4.00 6.38	0.65 1.75 3.70	2.80 4.50 6.78
Polonus	0.00 ^#^	0.14 0.75 2.24	0.69 2.00 4.38	2.49 4.25 6.69	1.79 3.50 6.06	4.61 6.75 9.47
Tisel	0.62 2.00 4.64	0.24 1.00 2.64	1.62 3.50 6.45	2.30 4.00 6.38	1.96 3.75 6.39	4.32 6.40 9.06
PC-110	0.37 1.50 3.89	0.78 2.00 4.11	0.43 1.50 3.64	3.65 5.75 8.53	2.69 4.75 7.66	3.99 6.00 8.58
Tiben	1.37 3.25 6.42	0.78 2.00 4.11	2.13 4.25 7.44	2.49 4.25 6.69	2.14 4.00 6.71	3.79 5.75 8.29
Nr 7/15	1.67 3.75 7.11	0.78 2.00 4.11	3.03 5.50 9.05	2.87 4.75 7.31	2.69 4.75 7.66	2.22 3.75 5.86
Gofert	0.90 2.50 5.37	0.36 1.25 3.02	2.31 4.50 7.77	2.49 4.25 6.69	2.32 4.25 7.03	6.27 8.75 11.81
Ores	1.99 4.25 7.78	0.49 1.50 3.39	2.85 5.25 8.73	3.06 5.00 7.62	2.51 4.50 7.35	6.48 9.00 12.10

^#^ Some of the genotypes have no confidence intervals because too few counts for them resulted in unstable models.

**Table 4:** Estimated mean number of larvae from four five-shoot replications per genotype. The cells show estimates with 95% confidence intervals estimated using generalized linear models. The genotypes sorted are according to the increasing mean probability across the three years and both pest generations.

Genotype	Estimated Mean Number of Larvae per Five-Shoot Replication (Estimates with 95% Confidence Intervals)					
	First Generation			Second Generation		
	2012	2013	2014	2012	2013	2014
Big Ben	0.00 0.25 1.91	1.50 3.50 6.78	0.09 0.75 2.58	2.62 4.00 5.80	3.50 4.75 6.26	3.11 5.00 7.54
Nr 8/72	0.67 2.25 5.36	0.26 1.25 3.50	1.71 3.75 6.99	3.23 4.75 6.69	4.36 5.75 7.40	1.08 2.25 4.07
Ben Connan	0.42 1.75 4.59	0.37 1.50 3.89	1.68 3.70 6.93	2.82 4.25 6.10	3.50 4.75 6.26	3.11 5.00 7.54
Foxendown	1.22 3.25 6.81	1.04 2.75 5.74	2.21 4.50 7.99	2.62 4.00 5.80	4.36 5.75 7.40	1.96 3.50 5.68
Fariegh	2.16 4.75 8.88	2.68 5.25 9.10	2.74 5.25 8.97	3.23 4.75 6.69	3.08 4.25 5.69	3.70 5.75 8.45
Ruben	2.16 4.75 8.88	1.66 3.75 7.12	1.39 3.25 6.32	5.54 7.50 9.88	5.46 7.00 8.81	4.30 6.50 9.35
Ben Alder	3.35 6.50 11.18	1.50 3.50 6.78	1.87 4.00 7.33	6.18 8.25 10.74	4.80 6.25 7.97	3.11 5.00 7.54
Ben Tirran	3.00 6.00 10.53	3.75 6.75 11.03	3.46 6.25 10.24	4.05 5.75 7.86	4.58 6.00 7.68	3.11 5.00 7.54
Polares	3.35 6.50 11.18	3.39 6.25 10.39	3.53 6.35 10.37	5.11 7.00 9.31	5.02 6.50 8.25	3.78 5.85 8.57
Ben Nevis	2.66 5.50 9.87	3.39 6.25 10.39	3.10 5.75 9.61	5.75 7.75 10.17	5.24 6.75 8.53	4.91 7.25 10.24
Ben Hope	2.16 4.75 8.88	3.75 6.75 11.03	3.10 5.75 9.61	6.18 8.25 10.74	5.68 7.25 9.09	4.50 6.75 9.64
Ojebyn	3.35 6.50 11.18	3.94 7.00 11.34	3.46 6.25 10.24	5.96 8.00 10.45	5.68 7.25 9.09	3.90 6.00 8.75
Ben Finlay	3.89 7.25 12.15	3.03 5.75 9.75	4.20 7.25 11.50	5.32 7.25 9.60	5.90 7.50 9.37	4.50 6.75 9.64
Nr 7/15	4.25 7.75 12.79	3.03 5.75 9.75	2.92 5.50 9.29	6.18 8.25 10.74	6.57 8.25 10.2	5.40 7.85 10.95
Tisel	2.16 4.75 8.88	3.94 7.00 11.34	4.47 7.60 11.94	6.18 8.25 10.74	6.12 7.75 9.64	5.53 8.00 11.12
Polonus	– 0.00 ^#^ –	0.26 1.25 3.50	1.71 3.75 6.99	7.08 9.25 11.82	5.68 7.25 9.09	5.73 8.25 11.41
Ben Lomond	4.43 8.00 13.10	4.12 7.25 11.66	3.83 6.75 10.88	5.75 7.75 10.17	5.24 6.75 8.53	4.91 7.25 10.24
PC-110	5.36 9.25 14.67	5.07 8.50 13.21	5.35 8.75 13.36	6.83 9.00 11.59	5.90 7.50 9.37	5.11 7.50 10.53
Gofert	2.16 4.75 8.88	4.69 8.00 12.59	5.74 9.25 13.97	7.26 9.50 12.15	7.01 8.75 10.76	7.62 10.50 14.03
Tiben	5.74 9.75 15.30	4.12 7.25 11.66	4.96 8.25 12.74	7.04 9.25 11.87	7.23 9.00 11.03	5.11 7.50 10.53
Polben	6.12 10.25 15.92	3.03 5.75 9.75	4.96 8.25 12.74	7.26 9.50 12.15	7.23 9.00 11.03	6.57 9.25 12.58
Ores	5.36 9.25 14.67	4.69 8.00 12.59	5.15 8.50 13.05	7.92 10.25 13.00	7.46 9.25 11.31	6.99 9.75 13.16

^#^ For genotype Polonus in 2012 in the first generation, no larvae were found, so this scenario was removed from the statistical analysis.

## Data Availability

Data is contained within the article.
